# The *Arabidopsis NLP7* gene regulates nitrate signaling via *NRT1.1*–dependent pathway in the presence of ammonium

**DOI:** 10.1038/s41598-018-20038-4

**Published:** 2018-01-24

**Authors:** Lufei Zhao, Wenjing Zhang, Yi Yang, Zehui Li, Na Li, Shengdong Qi, Nigel M. Crawford, Yong Wang

**Affiliations:** 1 State Key Laboratory of Crop Biology, College of Life Sciences, Shandong Agricultural University, Tai’an, Shandong 271018 China; 2 Section of Cell and Developmental Biology, Division of Biological Sciences, University of California at San Diego, La Jolla, California 92093-0116 USA

## Abstract

Nitrate is not only an important nutrient but also a signaling molecule for plants. A few of key molecular components involved in primary nitrate responses have been identified mainly by forward and reverse genetics as well as systems biology, however, many underlining mechanisms of nitrate regulation remain unclear. In this study, we show that the expression of *NRT1.1*, which encodes a nitrate sensor and transporter (also known as *CHL1* and *NPF6.3*), is modulated by NIN-like protein 7 (NLP7). Genetic and molecular analyses indicate that *NLP7* works upstream of *NRT1.1* in nitrate regulation when NH_4_^+^ is present, while in absence of NH_4_^+^, it functions in nitrate signaling independently of *NRT1.1*. Ectopic expression of *NRT1.1* in *nlp7* resulted in partial or complete restoration of nitrate signaling (expression from nitrate-regulated promoter NRP), nitrate content and nitrate reductase activity in the transgenic lines. Transcriptome analysis revealed that four nitrogen-related clusters including amino acid synthesis-related genes and members of *NRT1/PTR* family were modulated by both *NLP7* and *NRT1.1*. In addition, ChIP and EMSA assays results indicated that NLP7 may bind to specific regions of the *NRT1.1* promoter. Thus, *NLP7* acts as an important factor in nitrate signaling via regulating *NRT1.1* under NH_4_^+^ conditions.

## Introduction

Nitrogen is a vital macronutrient for plant growth and development. Plants have evolved a range of mechanisms to adapt to imbalanced nitrogen conditions. In agricultural systems, high-yield of crops relies on application of nitrogen fertilizers. But a large part of nitrogen deposited in the soil can’t be absorbed by plants and is lost to the environment, resulting in severe environmental and ecological pollution^1,2^. Improving the nitrogen use efficiency (NUE) of crops is the key to solve these problems. Studying the genes and mechanisms involved in regulating nitrogen uptake and assimilation can be a prerequisite for improving NUE of crops, therefore it is of great importance for sustaining agriculture. Nitrate and ammonium are the main nitrogen forms used by plants and most crops, like maize and wheat, take up nitrate as the major nitrogen source^3^. In addition to its nutrient role, nitrate acts also as a signaling molecule for plants. It regulates the expression levels of many genes, including genes directly involved in nitrate assimilation, namely *NIAs*, *NiR*, and some *NRTs* (short-term processes)^4–10^. It is also involved in many adaptive responses of plants^11^, such as root development and architecture, seed dormancy, flowering time, circadian system, leaf development, stomatal movements, and auxin transportation (long-term processes)^12–21^.

Nitrate is taken up into plants by nitrate transporters and high affinity and low affinity nitrate uptake systems have been identified^3^. Four gene families have been identified that encode nitrate transporters in *Arabidopsis*: *NRT1/PTR* (NPF, 53 members), *NRT2* (7 members), *CLC* (7 members), and *SLAC1/SLAH* (5 members)^22^. Among these families, NRT1/PTR belongs to the low affinity transport system, and NRT2 belongs to the high affinity transport system^22^. *NRT1.1* (also known as *CHL1* and *NPF6.3*), which belongs to NRT/PTR family, functions in nitrate uptake as both high affinity and low affinity transporter^23^.

In addition to the nitrate transport systems, genes involved in nitrate signaling have also been identified. Most of these genes were found to function in root architecture or primary nitrate responses^14,24,25^. The genes functioning in root architecture include the ANR1, the first molecular component isolated by classic molecular genetics approach^26^, is a MADS box transcription factor and positively regulates lateral root branching under sufficient nitrate condition^27–29^. *miR393/AFB3* and *NAC4* have been demonstrated to regulate the root system architecture in nitrate signaling using systems approach^26,30–32^. The split-root assays indicated that *TCP20* was involved in systemic nitrate signaling for root foraging^33^. Recently, TCP20 was found to regulate root meristem growth under nitrogen starvation and to interact with NLP6&7^34^. HHO1 and HRS1 are two nitrate-responsive transcription factors isolated by genome-wide analyses^35^. They function in the repression of primary root growth under both phosphate starvation and nitrate supply conditions.

During last several years, the nitrate regulatory factors involved in the primary nitrate response have been identified. NRT1.1, in addition to its transport function, was identified to work as a nitrate sensor^24,36–39^. The study on the crystal structure of NRT1.1 has demonstrated that Thr101 phosphorylation is essential for nitrate transport rate and provides further insights into its transport mechanisms^40–42^. CIPK8 and CIPK23 which belong to CBL-interacting protein kinase family^36,43^ are important players in responding to primary nitrate. CIPK8 works positively while CIPK23 functions negatively in nitrate regulation^36,43^. The expression of both *CIPK8* and *CIPK23* is regulated by *NRT1.1*^36,43^. Recently, *NRG2* which is an essential nitrate regulatory gene was isolated by forward genetics screen^44^. NRG2 acts as a positive nitrate regulatory factor and modulates *NRT1.1* expression and can interact with NLP7^44^. Additionally, several transcription factors were identified to be involved in primary nitrate response, for example, NLP6, NLP7, LBD37/38/39, TGA1, TGA4, and SPL9^10,45–49^. NLP7 is NIN-like protein and acts as an important nitrate positive regulator. NLP7 was isolated by reverse genetics strategy and the *nlp7* mutants exhibit a nitrogen-starved phenotype^46^. The nitrate condition can affect the NLP7′s nuclear retention^50^. Previous studies have demonstrated that the nitrate response *cis*-element NRE can be bound by NLPs^46,47^ and contain a DNA-binding domain RWP-PK and protein-protein interaction domains typeI/II Phox and Ben1p (PB1)^34,51^. ChIP-chip assays showed that NLP7 could bind 851 genes containing *NRT1.1*, *NRT2.1*, *LBD37/38*^50^. In addition, overexpression of *NLP7* can increase plant biomass, nitrogen uptake, total nitrogen content, and expression levels of genes involved in nitrogen assimilation and signaling^52^. Moreover, NLP7 can control plant root growth under both N-limited and N-rich conditions^46,52^. NLP6 also functions positively in nitrate regulation, is retained in the nucleus in nitrate-treated plants^34^ and can activate the expression of nitrate-responsive genes^47^. LBD37/38/39 are negative regulators in nitrate signaling. They are involved in primary nitrate response and can affect nitrogen status, growth, and nitrogen-dependent shoot branching^26,45^. *TGA1*, *TGA4*, and *SPL9* were isolated by systems approach^10,48,49^. TGA1 and TGA4 belong to bZIP transcription factor family and TGA1 can bind to the promoters of *NRT2.1* and *NRT2.2*^48^. SPL9 is demonstrated to be a nitrate regulatory hub^10^.

Although these nitrate regulatory genes have been identified, our understanding of the nitrate regulatory gene network is still incomplete. For example, both *NLP7* and *NRT1.1* play essential roles in regulating nitrate signaling and ChIP-chip assay showed that NLP7 might bind *NRT1.1*, however, their relationship and underlining mechanism remain unclear. In this paper, we investigated the relationship between *NRT1.1* and *NLP7* in nitrate regulation. Our analyses reveal that *NLP7* acts as a positive regulatory factor upstream of *NRT1.1* when NH_4_^+^ is present and modulates the nitrate signaling function of *NRT1.1*. *NLP7* might function in another pathway to regulate nitrate signaling independent of *NRT1.1*. In addition, transcriptome data showed that four GO terms related to nitrogen were regulated by *NRT1.1* as well as *NLP7* in nitrate signaling, providing more evidence to support our above conclusion. Furthermore, the ChIP and EMSA assays indicated that NLP7 could bind to specific regions of the *NRT1.1* promoter. Our findings not only further elucidate the relationship between *NRT1.1* and *NLP7*, but also provide insights into the network of the nitrate regulatory genes.

## Results

### The expression levels of *NRT1.1* are modulated by *NLP7*

**Figure 1:**
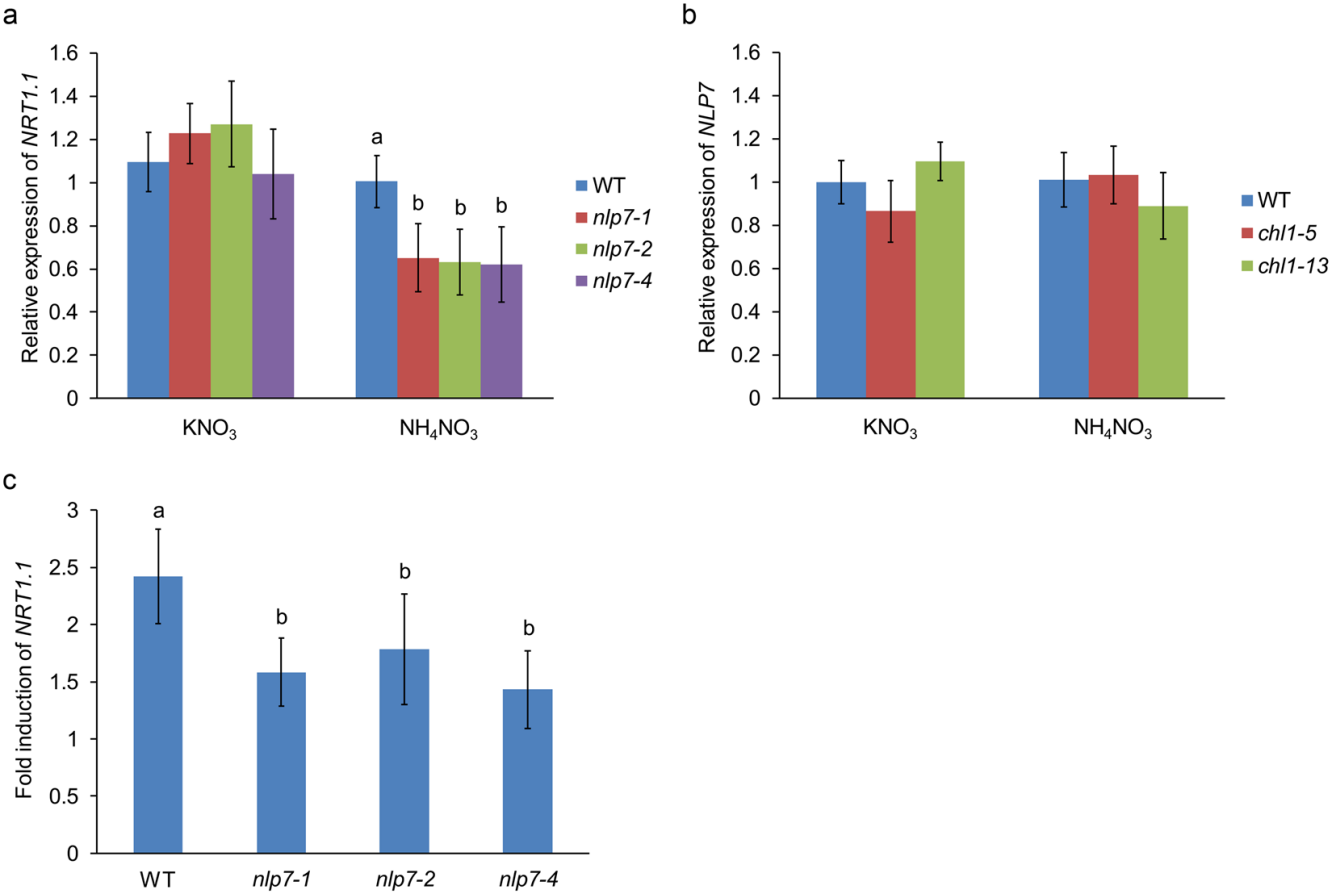
The transcript levels of *NRT1.1* in the *nlp7* plants were reduced when NH_4_^+^ is present. (**a**) Relative expression of *NRT1.1* in WT and the *nlp7* plants. Seedlings were planted on medium containing 10 mM potassium nitrate or 10 mM ammonium nitrate for 7d for testing *NRT1.1*′s expression. Error bars mean ± SD of four biological replicates. The significant difference (*p* < 0.05, t test) is indicated by different letters. (**b**) Relative expression of *NLP7* in WT and the *chl1* mutants. The growth conditions of plants are the same as (**a**). Error bars mean ± SD of four biological replicates. (**c**) Nitrate response of *NRT1.1* in WT and the *nlp7* mutants. Seedlings were planted on medium with 2.5 mM ammonium succinate for 7d, then treated with 10 mM KNO_3_ or KCl for 2 h followed by collecting samples for detecting nitrate induction of *NRT1.1*. Error bars mean ± SD of four biological replicates. The significant difference (*p* < 0.05, t test) is indicated by different letters.

To study the relationship between *NLP7* and *NRT1.1*, the expression levels of *NRT1.1* was detected firstly under potassium nitrate and ammonium nitrate conditions. Figure 1a showed that the transcript levels of *NRT1.1* in the *nlp7* mutants (*nlp7-1*, *nlp7-2*, and *nlp7-4*) were not notably changed under potassium nitrate condition, but was significantly decreased in mutant plants under ammonium nitrate condition (Fig. 1a). This indicates that the expression levels of *NRT1.1* can be modulated by *NLP7* in the presence of NH_4_^+^. In order to test if *NLP7* is regulated by *NRT1.1*, we tested *NLP7* expression in *chl1-5* and *chl1-13* mutants (the *nrt1.1* mutants) in potassium nitrate and ammonium nitrate mediums. The expression of *NLP7* was not changed in the *nrt1.1* mutants (Fig. 1b). This result indicates that *NRT1.1* may not regulate the expression of *NLP7*. We also tested the *NRT1.1* expression response to nitrate in WT and the *nlp7* mutants. qPCR results showed that the induction of *NRT1.1* by nitrate was notably decreased in the *nlp7* mutants, indicating that *NLP7* affects the response of *NRT1.1* to nitrate (Fig. 1c).

### *NLP7* and *NRT1.1* may participate in nitrate signaling in the same pathway

**Figure 2:**
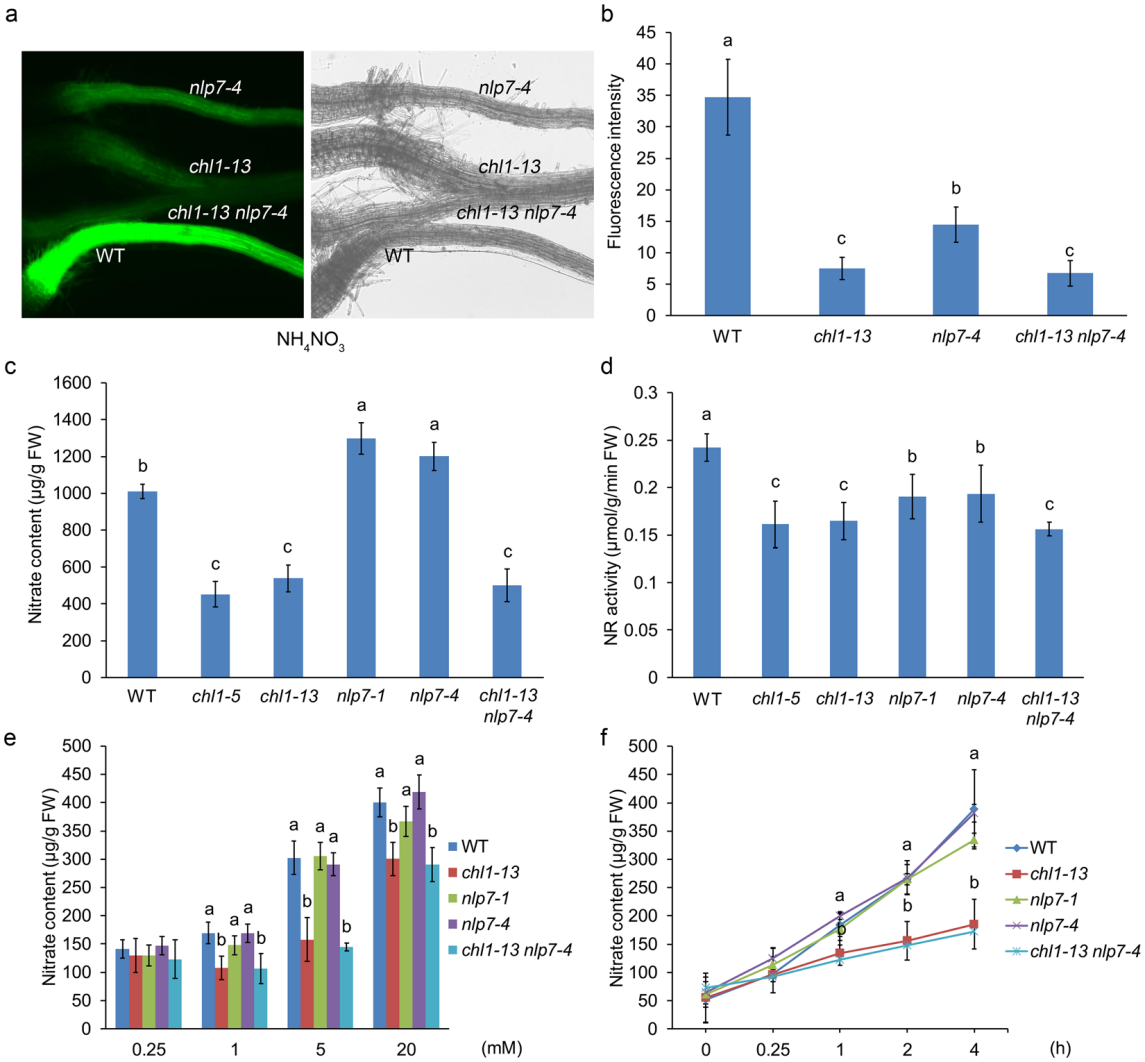
*NLP7* functions in the same nitrate regulation pathway as *NRT1*.*1*. (**a**) Root YFP observation of WT, *chl1-13*, *nlp7-4*, and *chl1-13 nlp7-4* plants. Fluorescence and light images of seedlings which grown on the medium with 10 mM ammonium nitrate for 5d were captured using a fluorescence microscope. (**b**) Quantification of root YFP signal levels of WT, *chl1-13*, *nlp7-4*, and *chl1-13 nlp7-4* plants. The same conditions for plants growth were used as (**a**). Error bars mean ± SD of sixty biological replicates. The significant difference (*p* < 0.05, t test) is indicated by different letters. (**c**) Nitrate content in WT, *chl1*, *nlp7*, and *chl1-13 nlp7-4* plants. Seedlings grown on the medium with 10 mM ammonium nitrate for 7d were sampled for nitrate content detection. Error bars mean ± SD of four biological replicates. The significant difference (*p* < 0.05, t test) is indicated by different letters. FW, fresh weight. (**d**) Nitrate reductase activity in WT, *chl1*, *nlp7*, and *chl1-13 nlp7-4* plants. The same growth conditions were used as (**c**). Error bars mean ± SD of four biological replicates. The significant difference (*p* < 0.05, t test) is indicated by different letters. FW, fresh weight. (**e**) and (**f**) Nitrate accumulation in WT, *nlp7*, *chl1-13* and *chl1-13 nlp7-4* plants. Seedlings were planted on the medium with 2.5 mM ammonium succinate for 7 d followed by the treatments with different KNO_3_ concentrations for 2 h (**e**) or with 5 mM KNO_3_ for different times (**f**). The whole plants were used for nitrate content test. Error bars mean ± SD of four biological replicates. The significant difference (*p* < 0.05, t test) is indicated by different letters.

To elucidate the relationship between *NLP7* and *NRT1.1*, the single mutants: *nlp7-4* and *chl1-13* which contain the nitrate-responsive NRP-YFP transgene, both of which were isolated by our mutant screens described previously^39,44^ were crossed to obtain the double mutant *chl1-13 nlp7-4*. The YFP signal levels in the roots of WT, two single mutants, and double mutants were detected. The results showed that the YFP signal of the two single mutants under the ammonium nitrate condition was much weaker than that of WT while the double mutant *chl1-13 nlp7-4* exhibited significantly weaker YFP signal than the *nlp7-4* mutant and similar to the *chl1-13* mutant (Fig. 2a,b). At the same time, we detected the YFP signal of the plants under the potassium nitrate condition without NH_4_^+^. The results showed that the YFP levels of the double mutant *chl1-13 nlp7-4* were much lower than those of WT and the *chl1-13* mutant, and similar to those of the *nlp7-4* mutant (Supplementary Fig. S1). Remarkably, the YFP signal of the *chl1-13* mutant was mildly weaker than WT. This result is consistent with previous studies demonstrating that *NRT1.1* is a key player in the nitrate regulation when NH_4_^+^ is present and functions poorly when NH_4_^+^ is absent^39,44^. Because we don’t observe any additive effects in the double mutant, these evidences indicate that *NLP7* and *NRT1.1* may participate in nitrate signaling in the same pathway.

In order to find physiological evidences for the relationship between *NLP7* and *NRT1.1*, we investigated the nitrate content and nitrate reductase activity in plants. Figure 2c showed higher nitrate content in the *nlp7* mutants while lower in the *chl1* plants than that in WT (Fig. 2c). The double mutant *chl1-13 nlp7-4* exhibited significant lower nitrate content than that in WT and the *nlp7* plants, and similar to that in the *chl1* mutants (Fig. 2c). The nitrate reductase activity of the two single mutants was much lower than that of WT, and the double mutant *chl1-13 nlp7-4* showed similar nitrate reductase activity to the *chl1-13* mutant (Fig. 2d). In addition, we analyzed the nitrate content in whole seedlings after treatments with different nitrate concentrations (0.25 mM to 20 mM) for 2 h and with 5 mM KNO_3_ for different times (0 h to 4 h) under the ammonium succinate condition. The results showed that the *nlp7* mutants displayed the same nitrate content as WT, but lower in the *chl1-13* mutant than in WT under both conditions (Fig. 2e,f). The double mutant *chl1-13 nlp7-4* showed the same nitrate content as the *chl1-13* mutant (Fig. 2e,f). Because *NRT1.1* is epistatic to *NLP7* in these experiments, these results indicate that *NLP7* and *NRT1.1* may be involved in the same pathway and *NLP7* may regulate nitrate signaling upstream of *NRT1.1*.

### *NRT1.1* can restore the phenotypes of *nlp7-4*

**Figure 3:**
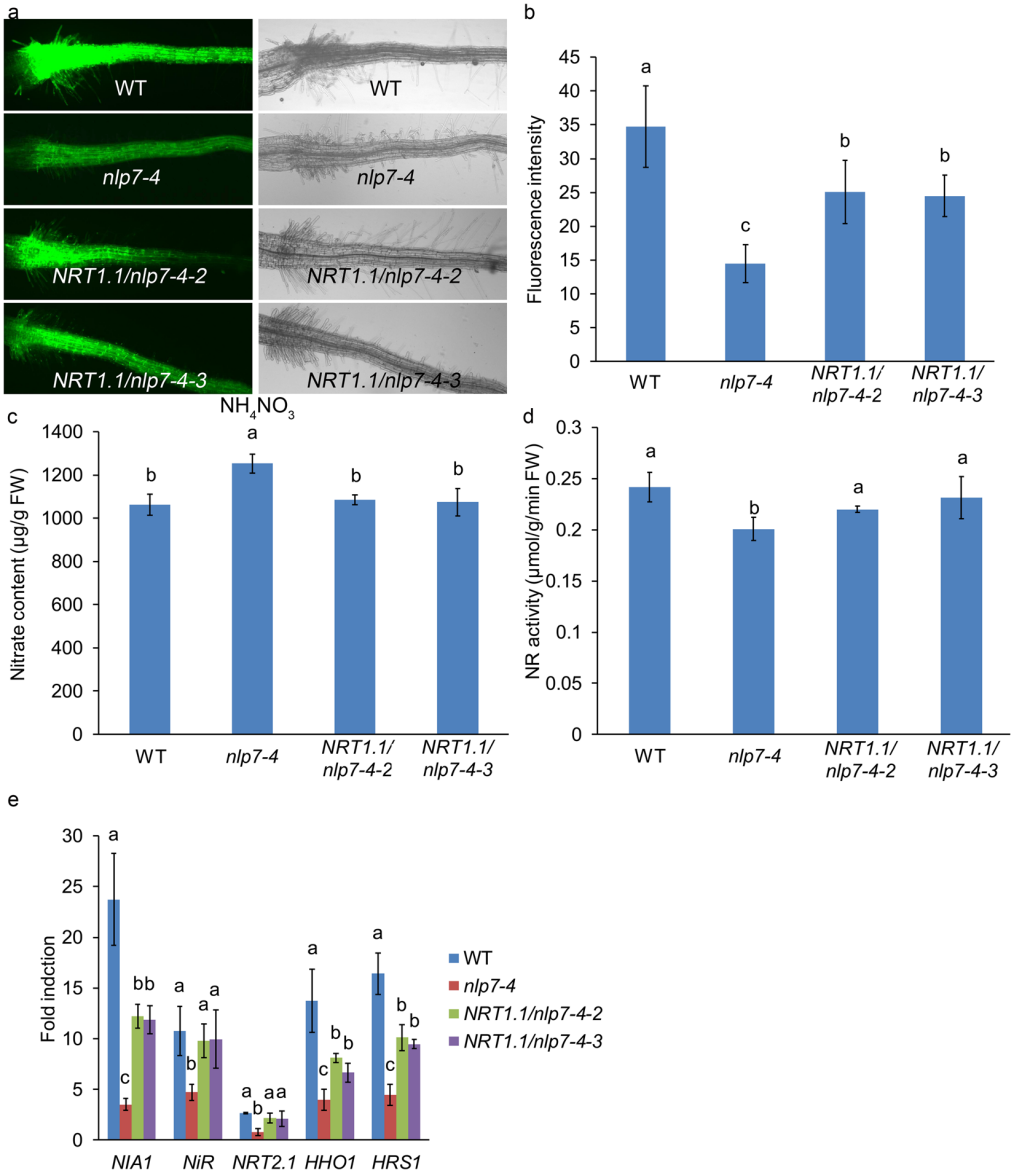
*NRT1.1* can restore the phenotypes of *nlp7-4*. (**a**) Root YFP observation of WT, *nlp7-4*, and *NRT1.1/nlp7-4* plants. Fluorescence and light images of seedlings which grown on the medium with 10 mM ammonium nitrate for 5d were captured using a fluorescence microscope. (**b**) Quantification of root YFP signal levels of WT, *nlp7-4*, and *NRT1.1/nlp7-4* plants. The same conditions for plants growth were used as (**a**). Error bars mean ± SD of sixty biological replicates. The significant difference (*p* < 0.05, t test) is indicated by different letters. (**c**) Nitrate content in WT, *nlp7-4*, and *NRT1.1/nlp7-4* plants. The 7-d-old seedlings on the medium with 10 mM ammonium nitrate were used for nitrate content detection. Error bars mean ± SD of four biological replicates. The significant difference (*p* < 0.05, t test) is indicated by different letters. FW, fresh weight. (**d**) Nitrate reductase activity in WT, *nlp7-4*, and *NRT1.1/nlp7-4* plants. The growth conditions are the same as (**c**). Error bars mean ± SD of four biological replicates. The significant difference (*p* < 0.05, t test) is indicated by different letters. FW, fresh weight. (**e**) The induction of endogenous genes after nitrate treatment in WT, *nlp7-4*, and *NRT1.1/nlp7-4* plants. Seedlings grown on 2.5 mM ammonium succinate medium for 7d were treated with 10 mM KNO_3_ or KCl for 2 h. Roots were used for detecting the expression of nitrate inducible genes by qPCR. Error bars mean ± SD of four biological replicates. The significant difference (*p* < 0.05, t test) is indicated by different letters.

In order to provide more evidence to support the conclusion that *NLP7* regulates nitrate signaling upstream of *NRT1.1*, we overexpressed *NRT1.1* in the *nlp7-4* mutant (Supplementary Fig. S2a). The transgenic lines *NRT1.1/nlp7-4* exhibited higher expression of *NRT1.1* than that in the WT and *nlp7* plants (Supplementary Fig. S2b). The YFP signal (from the NRP-YFP transgene) in the *NRT1.1/nlp7-4* plants grown under the ammonium nitrate condition was much stronger than that of the *nlp7-4* mutant, and weaker than that of WT (Fig. 3a). Quantifying the fluorescence intensity of the *NRT1.1/nlp7-4* plants showed that the YFP levels in the *NRT1.1/nlp7-4* plants were 70% higher than that in the *nlp7-4* mutant and reached 71% of that in WT (Fig. 3b). Then we tested the nitrate content and nitrate reductase activity in the *NRT1.1/nlp7-4* plants. As shown in Fig. 3c, the nitrate content in the *NRT1.1/nlp7-4* plants was lower than that in the *nlp7-4* mutant, and similar to that in WT (Fig. 3c). The nitrate reductase activity in the *NRT1.1/nlp7-4* plants was higher than that in the *nlp7-4* mutant, and reached the levels of WT (Fig. 3d).

In addition, we examined the transcript levels of *NIA1*, *NiR*, *NRT2.1*, *HHO1*, and *HRS1* which are responsive to nitrate in a short time. We found that the transcript levels of the nitrate inducible genes were significantly higher in the *NRT1.1/nlp7-4* plants than that in the *nlp7-4* mutant and completely or partially restored to the WT levels (Fig. 3e, Supplementary Fig. S3a), and there was no significant difference between WT and *NRT1.1/nlp7-4* plants before nitrate treatment (except for *NIA1*) (Supplementary Fig. S3b). Taken together, the phenotypes of *NRT1.1/nlp7-4* plants were completely or partially recovered to that of WT, further indicating that *NLP7* functions upstream of *NRT1.1*. Since *NRT1.1* functions in nitrate signaling pathway dependently on NH_4_^+^ while *NLP7* is NH_4_^+^ independent^44^, *NLP7* may also function in another nitrate signaling pathway independent on *NRT1.1* in the absence of NH_4_^+^.

### Transcriptome analysis of *nlp7* and *chl1* mutants

To obtain more comprehensive data on the relationship between *NLP7* and *NRT1.1*, we analyzed our transcriptome data of nitrate responses in the WT, *nlp7-4* and *chl1-13* plants. The experimental process, data quality, and the method of data filtering were described previously^44^.

Firstly, we compared the expression of nitrate inducible genes in the WT and the *chl1-13* mutant roots after nitrate treatment. The result showed that 152 genes could respond to nitrate in both WT and the *chl1-13* mutant (i.e. were unaffected by the *chl1-13* mutation, Supplementary Fig. S4a, Supplementary Table S1). In contrast, 438 genes (212 suppressed and 226 induced) lost their nitrate response in the *chl1-13* mutant. 47 genes (16 suppressed and 31 induced) showed a gain of nitrate response in the *chl1-13* mutant compared with WT. In summary, 485 genes were differentially expressed showed altered nitrate responses in the *chl1-13* mutant compared to WT.

We also compared the gene expression between WT and the *nlp7-4* mutant in response to nitrate. The results showed that there were 253 genes could respond to nitrate in both WT and the *nlp7-4* mutant (Supplementary Fig. S4b, Supplementary Dataset 2). The expression of 337 genes (158 down-regulated and 179 up-regulated) was responsive to nitrate in WT, but not in the *nlp7-4* mutant. 191 genes (102 down-regulated and 89 up-regulated) were responsive to nitrate exclusively in the *nlp7-4* mutant, but not in WT. In total, 528 genes were differentially expressed in the *nlp7-4* mutant.

**Figure 4:**
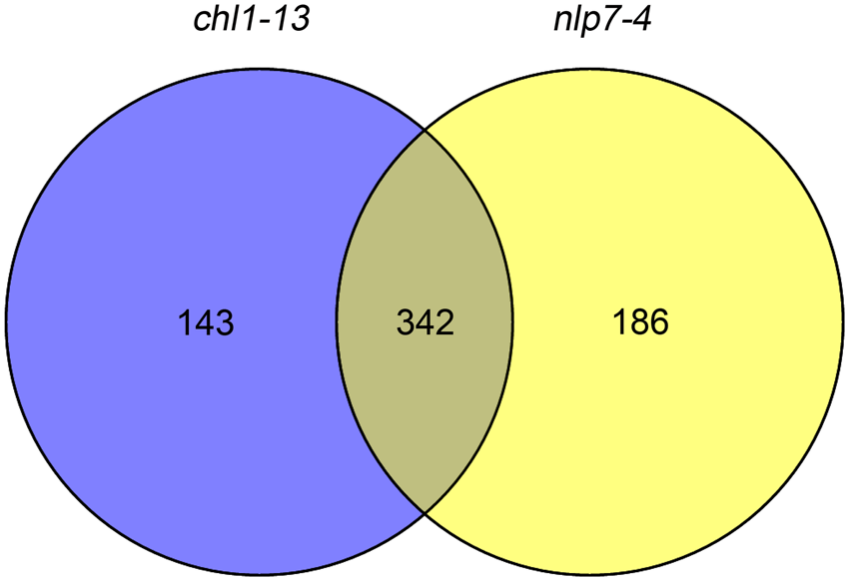
Venn diagram displaying the number of genes that are differentially expressed in *chl1-13* and *nlp7-4* mutant roots compared with WT. Seedlings were planted on the medium with 2.5 mM ammonium succinate for 7d followed by nitrate treatment.

We compared the 528 differentially expressed genes in the *nlp7-4* mutant and 485 differentially expressed genes in the *chl1-13* mutant and found 342 genes (64.8% of 528 and 70.5% of 485) were regulated by both *NLP7* and *NRT1.1* (Fig. 4, Supplementary Dataset 3) while 143 and 186 genes were regulated only by *NRT1.1* and *NLP7*, respectively (Fig. 4, Supplementary Dataset 4). Gene Ontology (GO) analysis of 342 shared genes showed four clusters related to nitrogen, including cellular amino acid biosynthetic process, cellular amino acid metabolic process, response to nitrogen compound, and nitrogen compound transport (Table 1). In these four clusters, genes involved in amino acid synthesis and nitrate transport were enriched (Supplementary Dataset 5). These data indicate extensive overlap in the target genes modulated by *NLP7* and *NRT1.1* and provide additional support that both *NLP7* and *NRT1.1* work in the same pathway in nitrate signaling.

**Table 1:** GO analysis for the nitrate responsive genes that are modulated by both *NRT1.1* and *NLP7*.

GO Term	*p* value
response to stimulus	3.61E-08
response to hormone	9.54E-06
response to biotic stimulus	1.02E-05
response to other organism	1.23E-05
response to organonitrogen compound	1.81E-05
response to chemical	2.50E-05
response to abiotic stimulus	2.53E-05
**cellular amino acid biosynthetic process**	**6.21E-05**
response to stress	6.61E-05
signal transduction	7.09E-05
**cellular amino acid metabolic process**	**1.74E-04**
response to auxin	2.53E-04
cellular response to stimulus	4.19E-04
intracellular signal transduction	5.11 E-04
monocarboxylic acid metabolic process	1.01E-03
**response to nitrogen compound**	**1.05E-03**
multi-organism process	1.32E-03
metabolic process	0.01542
**nitrogen compound transport**	**0.02518**

The 186 and 143 genes exclusively regulated by *NLP7* and *NRT1.1*, respectively (Supplementary Dataset 4) were further investigated by GO analysis. Among the 186 genes regulated by *NLP7*, there are two nitrogen-related clusters (response to nitrogen compound and nitrogen compound transport) (Table 2), including genes involved in amino acid transport and nitrate transport (Table 3). Among the 143 genes modulated by *NRT1.1*, three nitrogen-related clusters were found (response to nitrogen compound, cellular response to nitrogen compound, and response to nitrate) (Table 4). In the three clusters, gene involved in nitrate transport was enriched (Table 5). These data imply that *NLP7* may function in nitrate signaling pathway independent of *NRT1.1*.

**Table 2:** GO analysis for the nitrate responsive genes that are modulated only by *NLP7*.

GO Term	*p* value
ion transport	2.42E-05
transmembrane transport	4.70E-05
response to oxygen-containing compound	7.82E-05
response to endogenous stimulus	1.10E-04
cell communication	1.57E-04
response to acid chemical	1.66E-04
response to chemical	6.68E-04
single-organism process	9.50E-04
response to organic substance	1.02E-03
**response to nitrogen compound**	**1.41E-03**
response to hormone	1.86E-03
signaling	2.68E-03
oxidation-reduction process	3.20E-03
single-organism localization	3.54E-03
cellular response to endogenous stimulus	0.01051
cellular response to organic substance	0.01193
response to external stimulus	0.01199
**nitrogen compound transport**	**0.01332**
response to stimulus	0.01537
single-organism cellular process	0.01707

**Table 3:** Genes enriched in nitrogen compound response and transport clusters regulated by *NLP7*.

AGI	DESCRIPTION
AT5G47220	Ethylene responsive element binding factor 2; ERF2; ortholog
AT5G27100	Glutamate receptor 2.1; GLR2.1; ortholog
AT4G28140	At4g28140; At4g28140; ortholog
AT5G48410	Glutamate receptor 1.3; GLR1.3; ortholog
AT3G50930	Outer mitochondrial membrand protein of 66 kDa; OM66; ortholog
AT2G26530	At2g26530; AR781; ortholog
AT1G19020	CDP-diacylglycerol-glycerol-3-phosphate 3-phosphatidyltransferase; At1g19020; ortholog
AT4G18880	Heat shock transcription factor A4A; HSF A4A; ortholog
AT5G47230	Ethylene responsive element binding factor 5; ERF5; ortholog
AT3G01260	Galactose mutarotase-like superfamily protein; At3g01260; ortholog
AT3G16240	Delta tonoplast integral protein; Delta-TIP; ortholog
AT5G48430	Eukaryotic aspartyl protease family protein; At5g48430; ortholog
AT4G16370	Oligopeptide transporter; OPT3; ortholog
AT3G54830	Amino acid transporter; At3g54830; ortholog
AT5G24920	Glutamine dumper 5; GDU5; ortholog
AT5G04770	Cationic amino acid transporter 6; CAT6; ortholog
AT5G47450	Tonoplast intrinsic protein 2;3; TIP2;3; ortholog
AT1G12940	Nitrate transporter 2.5; NRT2.5; ortholog

**Table 4:** GO analysis for the genes regulated only by *NRT1.1* after nitrate treatment.

GO Term	*p* value
ammonia assimilation cycle	3.24E-06
glutamate biosynthetic process	9.02E-05
L-glutamate biosynthetic process	9.02E-05
glutamine metabolic process	1.31E-04
response to chemical	2.56E-04
response to stimulus	3.33E-04
response to organic substance	5.87E-04
**response to nitrogen compound**	**8.74E-04**
anion transport	0.0011
glutamine family amino acid metabolic process	0.0015
response to nutrient levels	0.0019
cellular response to inorganic substance	0.0021
ion transport	0.0024
oxoacid metabolic process	0.0033
organic acid metabolic process	0.0034
malate transport	0.0039
alpha-amino acid metabolic process	0.0040
**cellular response to nitrogen compound**	**0.0044**
response to starvation	0.0061
**response to nitrate**	**0.0110**
small molecule metabolic process	0.0169

**Table 5:** Genes involved in nitrogen compound response and nitrate response clusters regulated by *NRT1.1*.

AGI	DESCRIPTION
AT4G31910	BR-RELATED ACYLTRANSFERASE1; BAT1; ortholog
AT1G69440	A member of the ARGONAUTE family; AGO7; ortholog
AT2G30040	Member of MEKK subfamily; MAPKKK14; ortholog
AT2G18690	Transmembrane protein; At2g18690; ortholog
AT2G26150	member of Heat Stress Transcription Factor (Hsf) family; HSFA2; ortholog
AT3G01420	ALPHA-DIOXYGENASE 1; ALPHA-DOX1; ortholog
AT4G31710	GLUTAMATE RECEPTOR 2.4; GLR2.4; ortholog
AT5G14120	Major facilitator superfamily protein; At5g14120; ortholog
AT5G60770	NITRATE TRANSPORTER 2.4; NRT2.4; ortholog

### NLP7 can bind to the promoter of *NRT1.1*

**Figure 5:**
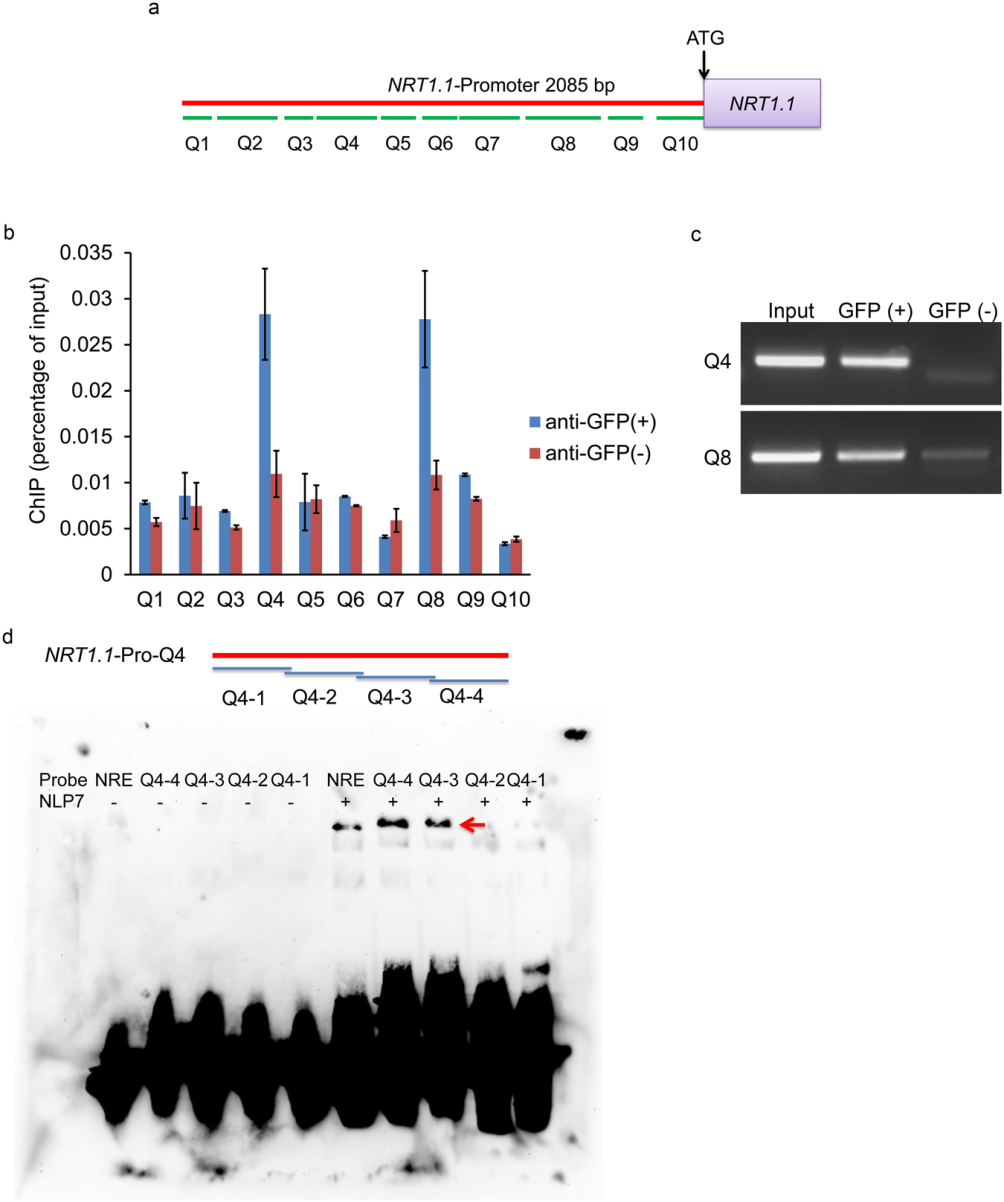
NLP7 binds to the promoter of *NRT1*.*1*. (**a**) Segmentation schematic diagram of the promoter of *NRT1.1*. Q1-Q10 represent different fragments of *NRT1*.*1* promoter. (**b**) ChIP-qPCR assay shows that NLP7 binds to *NRT1.1* promoter *in vivo*. ChIP assays were performed with *nlp7-1* seedlings expressing pNLP7:NLP7-GFP fusion protein grown on 10 mM ammonium nitrate medium for 7 d. A sample without antibody was used as the negative control for the ChIP-qPCR (n = 3). (**c**) ChIP-PCR assay shows that NLP7 binds to *NRT1.1* promoter *in vivo*. ChIP assays were performed with the same seedlings as in (**b**). Input was used as positive control and a sample without antibody was used as the negative control. Q4 and Q8 are the promoter sites of *NRT1.1* showing the binding activity with NLP7 and images come from the checking gel shown in the Supplementary Fig. 5. (**d**) EMSA assay shows that NLP7 binds to *NRT1.1* promoter *in vitro*. Red arrow indicated the positions of protein-DNA complexes caused by binding of NLP7 to DNA probes. The NRE probe was used as a positive control. The DNA probes were listed in Supplementary Dataset 6.

Previous ChIP-chip test indicated that *NRT1.1* was one of the NLP7 targets^50^. To investigate further the binding of NLP7 to *NRT1.1*, we carried out a ChIP assay using the transgenic plants expressing pNLP7:NLP7-GFP fusion protein in *nlp7-1* mutant. The ChIP-qPCR assay was performed using a series of primers designed to cover the *NRT1.1* promoter region (Fig. 5a) and a sample without antibody was used as a negative control. The results showed that NLP7-specific enrichments were much higher in two regions of *NRT1.1* promoter in the sample than in negative control, and the two regions were −1537 to −1334bp, and −686 to −465bp, respectively (Fig. 5b). ChIP-PCR results confirmed the binding of NLP7 to the two regions (Fig. 5c, Supplementary Fig. 5). These results indicate that NLP7 can bind to the promoter of *NRT1.1 in vivo*. We also did the ChIP-qPCR and ChIP-PCR assays with the seedlings expressing p35S::NLP7-GFP fusion protein in *nlp7-1* plant. The results also showed that NLP7 could bind to *NRT1.1* promoter *in vivo* (Supplementary Fig. S6b,c). To provide more evidences to the binding of NLP7 to *NRT1.1* promoter, electrophoretic mobility shift assay (EMSA) was performed. The result showed that NLP7 could bind to the Q4-3 and Q4-4 regions of *NRT1.1* promoter *in vitro* (Fig. 5d), implying that NLP7 may bind to the specific regions of *NRT1.1* promoter. However, no activity was found for the binding of NLP7 to the Q8 region of *NRT1.1* promoter (Supplementary Fig. S7).

## Discussion

NRT1.1 have been characterized to be a nitrate transceptor in previous reports^36^ and can regulate the expression of nitrate regulatory genes *CIPK8* and *CIPK23*^36,43^. Moreover, NRT1.1 can be phosphorylated by CIPK23 at amino acid Thr-101 under low nitrate concentration^36^. NLP7 is another key nitrate regulator^46,47,50^. As a transcription factor, it can bind to the nitrate response *cis*-elements (NREs)^47^. ChIP-chip and ChIP-qPCR assays have illustrated that NLP7 binds to many genes functioning in the nitrate regulation and assimilation, for instance *NRT2.1*, *NRT2.2*, and *LBD37*^47,50^. It has been reported that NLP7 could bind *NRT1.1*, but it is not known if NLP7 binds to the promoter of and regulates *NRT1.1*.

In this paper, our results revealed that the transcript levels of *NRT1.1* were down-regulated in the *nlp7* plants when NH_4_^+^ is present. The nitrate induction of *NRT1.1* was reduced as well in the mutants (Fig. 1). These data indicate that the expression and induction of *NRT1.1* can be modulated by *NLP7* in the presence of NH_4_^+^. Our genetic analysis showed that the nitrate-responsive YFP signal, nitrate content, nitrate reductase activity, and nitrate uptake in the double mutant *chl1-13 nlp7-4* were strongly reduced than those in WT and close to those in *chl1-13* mutant (Fig. 2). These results indicate that *NLP7* participates in nitrate signaling in the same pathway as *NRT1.1* and *NLP7* may work upstream of *NRT1.1*. We also overexpressed the *NRT1.1* in the *nlp7-4* mutant and found that the YFP signal, nitrate content, nitrate reductase activity, and the induction levels of the endogenous genes after nitrate treatment in the *NRT1.1/nlp7-4* plants were completely or partially recovered to WT levels (Fig. 3). Even though the levels of *NRT1.1* mRNA were higher in the transgenic plants, one was similar to WT levels and both lines gave similar results. These results demonstrate that *NLP7* regulates nitrate signaling upstream of *NRT1.1*.

We found that the nitrate content was significant higher in the *nlp7* mutants than in WT (Fig. 2c), consistent with previous studies^46,52^. The reason for the higher nitrate content in the mutants may come from higher nitrate uptake and/or lower nitrate reduction. We tested the nitrate accumulation and found no significant difference between the *nlp7* mutants and WT (Fig. 2e,f), similar to the report that nitrate uptake activity of the *nlp7-1* mutant at 5 mM ^15^NO_3_^−^ external concentration was the same as that of WT^52^. We also tested the total nitrogen content and similar levels were found in the *nlp7* mutants and WT, indicating that the nitrate import was not changed in the *nlp7* plants (Supplementary Fig. S8). However, the nitrate reductase activity in the *nlp7* plants was much lower than that in WT (Fig. 2d). These findings indicate that the higher nitrate content in the *nlp7* mutants is caused by the reduced nitrate assimilation, rather than altered nitrate uptake. In addition, it has been reported that the nitrate content is lower and the nitrate uptake ability is reduced in *chl1* mutants. Interestingly, our data revealed that the nitrate reductase activity was notably depressed in the *chl1* mutants, indicating that the reduced nitrate content in the *chl1* mutants may result from both decreased nitrate uptake and reduction.

*NRT1.1* is an essential component of nitrate uptake and nitrate signaling, respectively^36^. But its function in nitrate signaling has been found to work mainly when NH_4_^+^ is present^39,44^. In this paper, we found that the expression of *NRT1.1* could be modulated by *NLP7* when NH_4_^+^ is present (Fig. 1a) and the nitrate uptake of *nlp7* mutants was not affected (Fig. 2e,f, Supplementary Fig. S8). Combining with our results that *NLP7* works upstream of *NRT1.1*, these findings indicate that *NLP7* regulates the nitrate signaling function but does not affect the transport function of *NRT1.1*. *NRG2*, another important nitrate regulatory gene, has been reported to modulate the expression of *NRT1.1* in both presence and absence of NH_4_^+^, indicating that *NRG2* can affect both nitrate transport and signaling functions of *NRT1.1*^44^. These findings demonstrate that both *NRG2* and *NLP7* can regulate *NRT1.1* but in different ways. *NRT1.1*′*s* functioning as a regulatory gene is NH_4_^+^-dependent, while *NLP7* is NH_4_^+^-independent and *NRT1.1* can restore the phenotype of the *nlp7-4* mutant (Fig. 3). Accordingly, these findings suggest that *NLP7* functions in nitrate signaling independent of *NRT1.1* in the absence of NH_4_^+^.

Our RNA-sequencing analysis showed that many genes were regulated by both *NLP7* and *NRT1.1* in response to nitrate (Fig. 4). GO analysis of these genes revealed that there were four nitrogen-related clusters, including cellular amino acid biosynthetic process, cellular amino acid metabolic process, response to nitrogen compound, and nitrogen compound transport, modulated by both *NLP7* and *NRT1.1* (Table 1). These data provide further evidences that *NLP7* modulates nitrate regulation in the same pathway as *NRT1.1* under these conditions.

Since NLP7 is a transcription factor and ChIP-chip test has shown that it could bind *NRT1.1*^50^, we performed ChIP-qPCR, ChIP-PCR, and EMSA to investigate the binding activity of NLP7 to the promoter of *NRT1.1*. These results indicate that NLP7 can bind to the promoter of *NRT1.1* (Fig. 5, Supplementary Figs S5, S6). ChIP assays showed that NLP7 could bind the Q4 and Q8 region of *NRT1.1* promoter (Fig. 5b,c, Supplementary Figs S5, S6), while EMSA assay revealed that NLP7 could bind to Q4 region, but not Q8 region of *NRT1.1* promoter (Fig. 5d, Supplementary Fig. S7), implicating that NLP7 may bind Q8 region indirectly.

These results were obtained from the conditions with both ammonium and nitrate. Since ammonium is crucial for the regulation of *NRT1.1* by *NLP7*, the presence of NH_4_^+^ might be also important for the binding of NLP7 to the promoter of *NRT1.1*. Without NH_4_^+^, the NLP7 protein may not bind to the promoter of *NRT1.1*, resulting in the lost regulation function of *NLP7* to *NRT1.1*.

**Figure 6:**
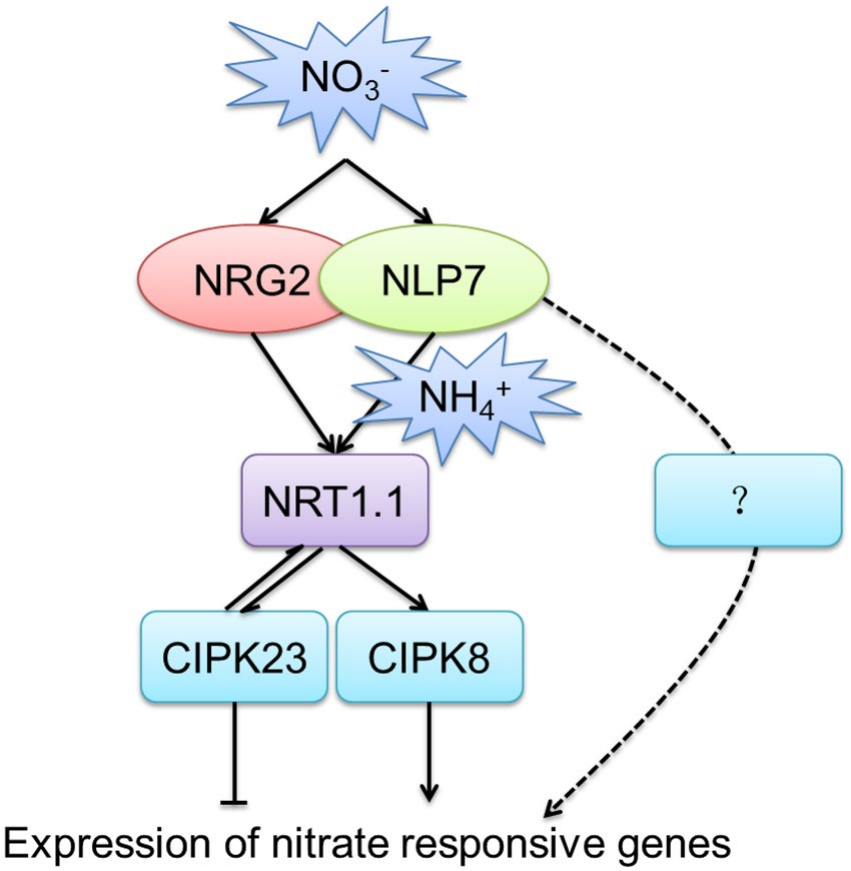
Proposed model for the nitrate regulatory genes in the nitrate signaling. *NLP7* regulates nitrate signaling upstream of *NRT1.1* when NH_4_^+^ is present and may regulate an *NRT1.1*-independent pathway in the absence of NH_4_^+^ in nitrate signaling. NLP7 interacts with NRG2 and *NRG2* works upstream of *NRT1.1* when NH_4_^+^ is present and/or absent. *NRT1.1* regulates *CIPK8* and *CIPK23* at the transcript level. CIPK23 can phosphorylate NRT1.1. These genes are all involved in regulating the expression of nitrate responsive genes.

Taken together, in the primary nitrate response, *NLP7* regulates nitrate signaling upstream of *NRT1.1* in the presence of NH_4_^+^ and may modulate an *NRT1.1*-independent pathway in the absence of NH_4_^+^ in nitrate signaling. Moreover, *NLP7* may regulate *NRT1.1* via binding to the promoter of *NRT1.1*. Therefore we propose the working model as shown in Fig. 6. *NLP7* plays a key role through regulating *NRT1.1* in the presence of NH_4_^+^ while through an *NRT1.1*-independent pathway in nitrate signaling when NH_4_^+^ is absent. NLP7 can interact with NRG2 and *NRG2* works upstream of *NRT1.1* when NH_4_^+^ is present and/or absent. *NRT1.1* regulates the transcript levels of *CIPK8* and *CIPK23* and can be phosphorylated by CIPK23 to switch its nitrate affinity. The genes regulated by *NLP7* in the nitrate signaling pathway without NH_4_^+^ need to be investigated. The characterization of the regulation relationship of *NLP7* and *NRT1.1* has provided insights into the regulatory mechanisms of the genes functioning in nitrate regulation.

## Methods

### Plant materials

*Arabidopsis thaliana* Col-0 was used as the ecotype of wild-type in this research Homozygous transgenic seeds with the NRP-YFP construct^39^ and carrying p35S::NLP7-GFP or pNLP7:NLP7-GFP^50^, the mutant lines *chl1-5*^36^, *chl1-13* containing the NRP-YFP construct^39^, *nlp7-1*, *nlp7-2*^46^, *nlp7-4* containing the NRP-YFP construct^44^ were described previously. Transgenic plants carrying *p35S::NRT1.1 cDNA-FLAG* were obtained by floral dipping^53^ of *nlp7-4* mutants.

### Growth and treatment conditions

To test gene expression in plants, seedlings grown on 10 mM ammonium nitrate or potassium nitrate medium (aseptic hydroponics) for 7 days were collected for qPCR analysis. To test YFP signal of the plants containing the NRP-YFP construct, seedlings were cultured on mediums containing 10 mM ammonium nitrate or potassium nitrate for 5 days. The fluorescence was observed under a fluorescence microscope (Nikon Eclipse Ti-S) and ImageJ was used to quantify the YFP signal levels of plants. For the nitrate uptake assays, seedlings were grown on medium containing 2.5 mM ammonium succinate (aseptic hydroponics) for 7 days followed by treatments with different nitrate concentrations (0.25 mM to 20 mM) for 2 h or with 5 mM KNO_3_ for different times (0 h to 4 h). For qPCR analysis of nitrate responsive genes, seedlings were cultured as described^44^. For nitrate content test and nitrate reductase activity assays, seedlings were cultured on medium containing 10 mM ammonium nitrate (aseptic hydroponics) for 7 days.

### qPCR analysis

Total RNA was extracted from plants using an ultrapure RNA kit (CWBIO). cDNA samples were carried out using a EasyScript One-Step gDNA Removal and cDNA Synthesis SuperMix Kit (TransGen Biotech). The UltraSYBR Green Mixture qPCR kit (CWBIO) was used in the qPCR reaction. The real-time PCR was carried out using VIIA7 (Applied Biosystems). We used *TUB2* (At5g62690) as the internal control.

### Nitrate content, nitrate reductase (NR) activity and total nitrogen content assays

Nitrate in the plants was extracted with the method as previously described^44^ and the samples were measured with the hydrazine-sulfate method^7,39^ using the machine AutoAnalyzer 3 (SEAL). Nitrate reductase activity was measured mainly based on the method described previously^54,55^. Briefly, about 0.1 g fresh samples were frozen by liquid nitrogen and broken into powder by a RETCH MM4000. 1 mL of extraction buffer (25 mM phosphate buffer, pH7.5, 5 mM cysteine, 5 mM EDTA) was added into the samples and then centrifuged at 4 °C and 4000 g for 5 min. 100 µL supernatant was transferred into a new 1.5-mL tube, then 375 µL 0.1 M KNO_3_-phosphate buffer and 125 µL 2 mg/mL NADH_2_ were added. The reactions were incubated at 25 °C for 30 min. After 250 µL of 1% sulfanilic acid and 250 µL of 0.2% α-naphthylamine were added and incubated at room temperature for 20 min, the OD_540_ value was measured. 100 µL of extraction buffer was used as a control. The following equation was used to calculate nitrate reductase activity: A = C × V_1_/V_2_/W/t (A, nitrate reductase activity; C, nitrite amount deduced from the regression equation; V_1_, the volume of extraction reagent; V_2_, volume of supernatant added into the reaction; W, sample weight; t, reaction time). The concentrations of NO_2_^−^ used in standard curve were at the amount from 0.00435 to 0.0435µmol and regression equation was deduced from the basis of standard curve. Total nitrogen content was tested with the machine AutoAnalyzer 3 (SEAL). Weighed samples (~0.1 g DW) were digested using H_2_SO_4_-H_2_O_2_ and the samples were measured using the machine AutoAnalyzer 3 (SEAL).

### Transcriptome analysis

The transcriptome analysis was performed using RNA sequencing technology. Methods for RNA sequencing assay and data filtering were described previously^44^. GO enrichment analysis of the data was performed using Omicshare (http://www.omicshare.com/tools/Home/Soft/gogsea). We have deposited the RNA sequencing data into National Center for Biotechnology Information database (accession number SRP067979, www.ncbi.nlm.nih.gov/sra)^44^.

### ChIP assay

The transgenic plants carrying NLP7-GFP were planted under 10 mM ammonium nitrate condition for 7 days, the whole seedlings were used for ChIP assay. The ChIP experiment was carried out following the procedure described previously^56,57^. All of the primers used in ChIP-qPCR or ChIP-PCR are listed in Supplementary Dataset 6.

### EMSA

The DNA probes were labelled by using Biotin 5′ and the DNA probes were listed in the Supplementary Dataset 6. The NLP7 protein was obtain by the transient transformation of *Nicotiana benthamiana* with the 35Spro:: NLP7-FLAG construct. The EMSA assays were performed using the Lightshift Chemiluminescent EMSA kit (Beyotime Biotechnology).

### Statistical analysis

Statistically significant differences were computed based on the Student’s t-tests.

## Electronic supplementary material

Supplementary Figure

Supplementary Dataset 1

Supplementary Dataset 2

Supplementary Dataset 3

Supplementary Dataset 4

Supplementary Dataset 5

Supplementary Dataset 6
