# Supplementary material for: The Arabidopsis NLP7 gene regulates nitrate signaling via NRT1.1–dependent pathway in the presence of ammonium
Source: Sci Rep. 2018 Jan 24;8:1487. doi: 10.1038/s41598-018-20038-4 (PMC5784019; doi:10.1038/s41598-018-20038-4)
Supplement: Supplementary file 1 — Supplementary Figure [file 41598_2018_20038_MOESM1_ESM.pdf]

The *Arabidopsis NLP7* gene regulates nitrate signaling via *NRT1.1*–dependent pathway in the presence of ammonium

Lufei Zhao[a], Wenjing Zhang[a], Yi Yang[a], Zehui Li,[a] Na Li[a], Shengdong Qi[a], Nigel M. Crawford[b], and Yong Wang[a,1]

[a]State Key Laboratory of Crop Biology, College of Life Sciences, Shandong Agricultural University, Tai'an, Shandong 271018, China

[b]Section of Cell and Developmental Biology, Division of Biological Sciences, University of California at San Diego, La Jolla, California 92093-0116, USA

[1]Corresponding author: Yong Wang

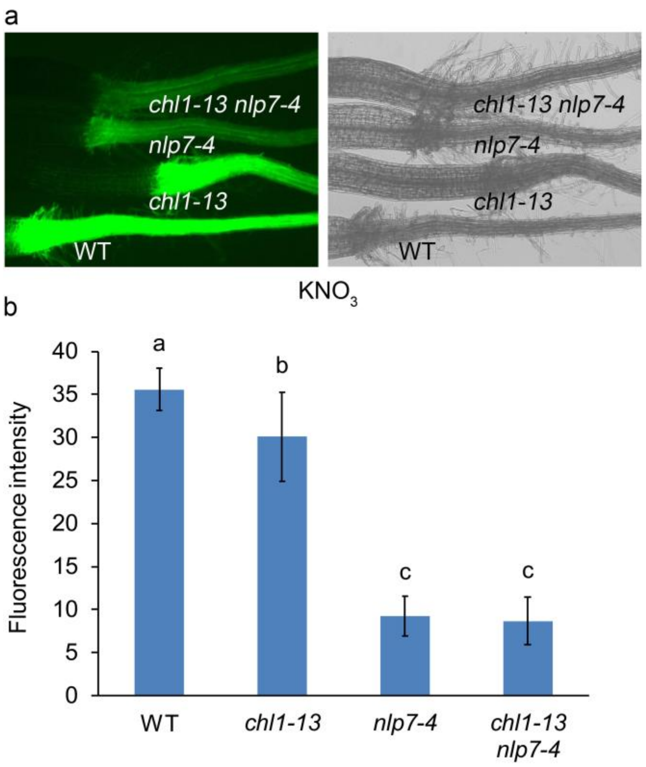

Figure S1. The roots fluorescence of *chl1-13 nlp7-4* was similar to that of *nlp7-4*. (a) Root YFP observation of WT, *chl1-13*, *nlp7-4*, and *chl1-13 nlp7-4* plants. Fluorescence and light images of seedlings which grown on the medium with 10mM potassium nitrate for 5d were captured used a fluorescence microscope. (b) Quantification of root YFP signal levels of WT, *chl1-13*, *nlp7-4*, and *chl1-13 nlp7-4* plants. The growth conditions of plants are the same as (a). Error bars mean ± SD of sixty biological replicates. Different letters indicate the significant difference (p<0.05, t test).

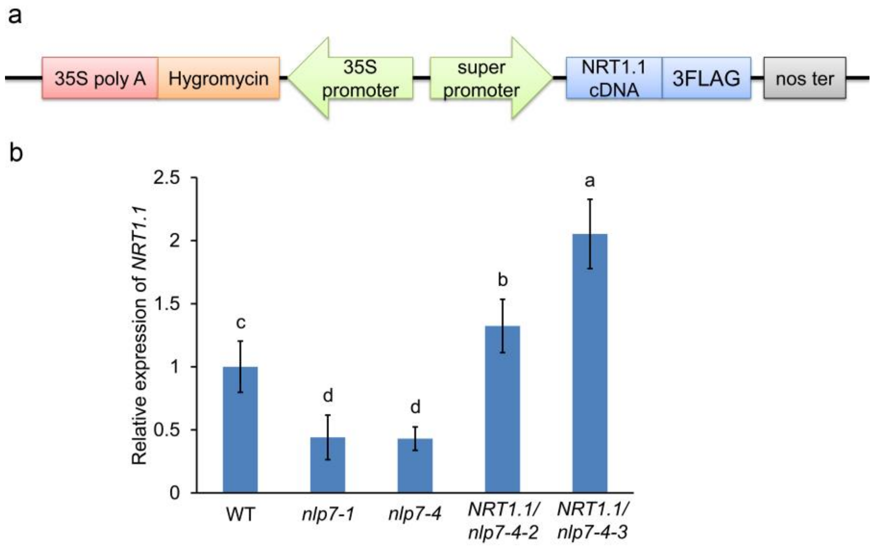

Figure S2. Identification of the transgenic lines *NRT1.1/nlp7-4*. (a) Schematic diagram of the T-DNA region of the expression vector pSUPER1300-*NRT1.1*. (b) The expression levels of *NRT1.1* in wild type, *nlp7*, and the transgenic lines *NRT1.1/nlp7-4*. Seedlings grown on 10mM ammonium nitrate medium for 7d were collected for qPCR analysis. Error bars mean ± SD of four biological replicates. Different letters indicate the significant difference (p<0.05, t test).

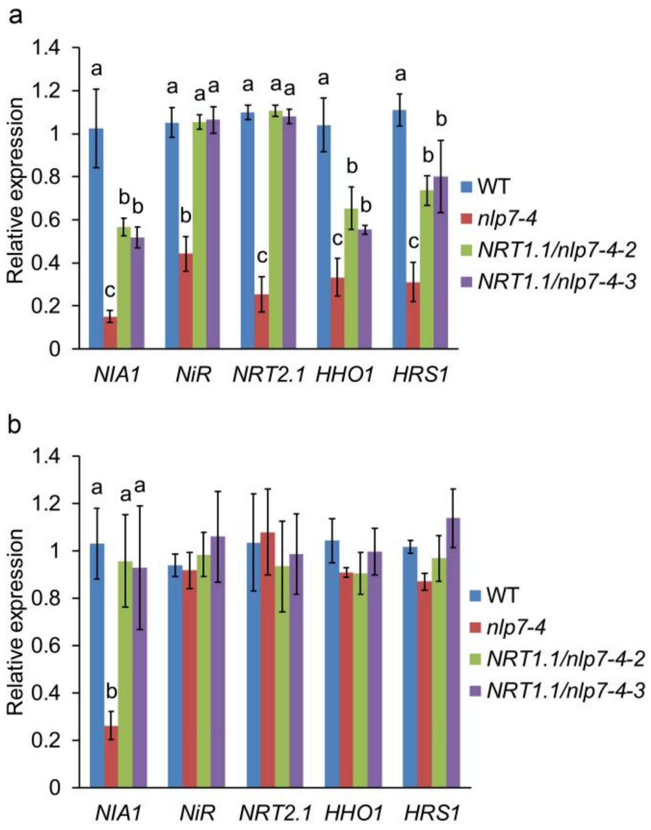

Figure S3. The expression of nitrate responsive genes in *NRT1.1/nlp7-4* was similar to wild type. (a) The expression of nitrate responsive genes in wild type, *nlp7-4*, and *NRT1.1/nlp7-4* plants. Seedlings were grown on 2.5mM ammonium succinate medium for 7d were treated with KNO_3 for 2h. Roots were collected for RNA extraction and the expression of nitrate responsive genes was detected by qPCR. (b) The expression of nitrate responsive genes in wild type, *nlp7-4*, and *NRT1.1/nlp7-4* plants. Seedlings were grown on 2.5mM ammonium succinate medium for 7d were treated with KCl for 2h. Roots were collected for RNA extraction and the expression of nitrate responsive genes was detected by qPCR. Error bars mean ± SD of four biological replicates. Different letters indicate the significant difference (p<0.05, t test).

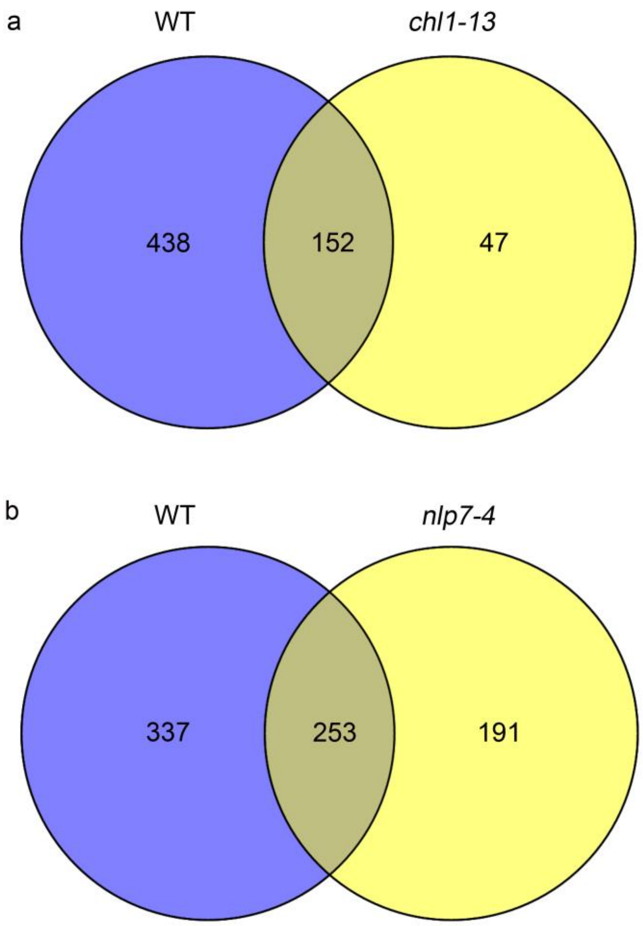

Figure S4. Venn diagram showing the number of genes differentially expressed in roots of wild type, *chl1-13* and *nlp7-4* mutants. (a) Venn diagram showing the number of genes regulated by nitrate in wild type and *chl1-13* mutant. Seedlings were grown on 2.5mM ammonium succinate followed by nitrate treatment. (b) Venn diagram showing the number of genes regulated by nitrate in wild type and *nlp7-4* mutant. Seedlings were grown on ammonium succinate followed by nitrate treatment.

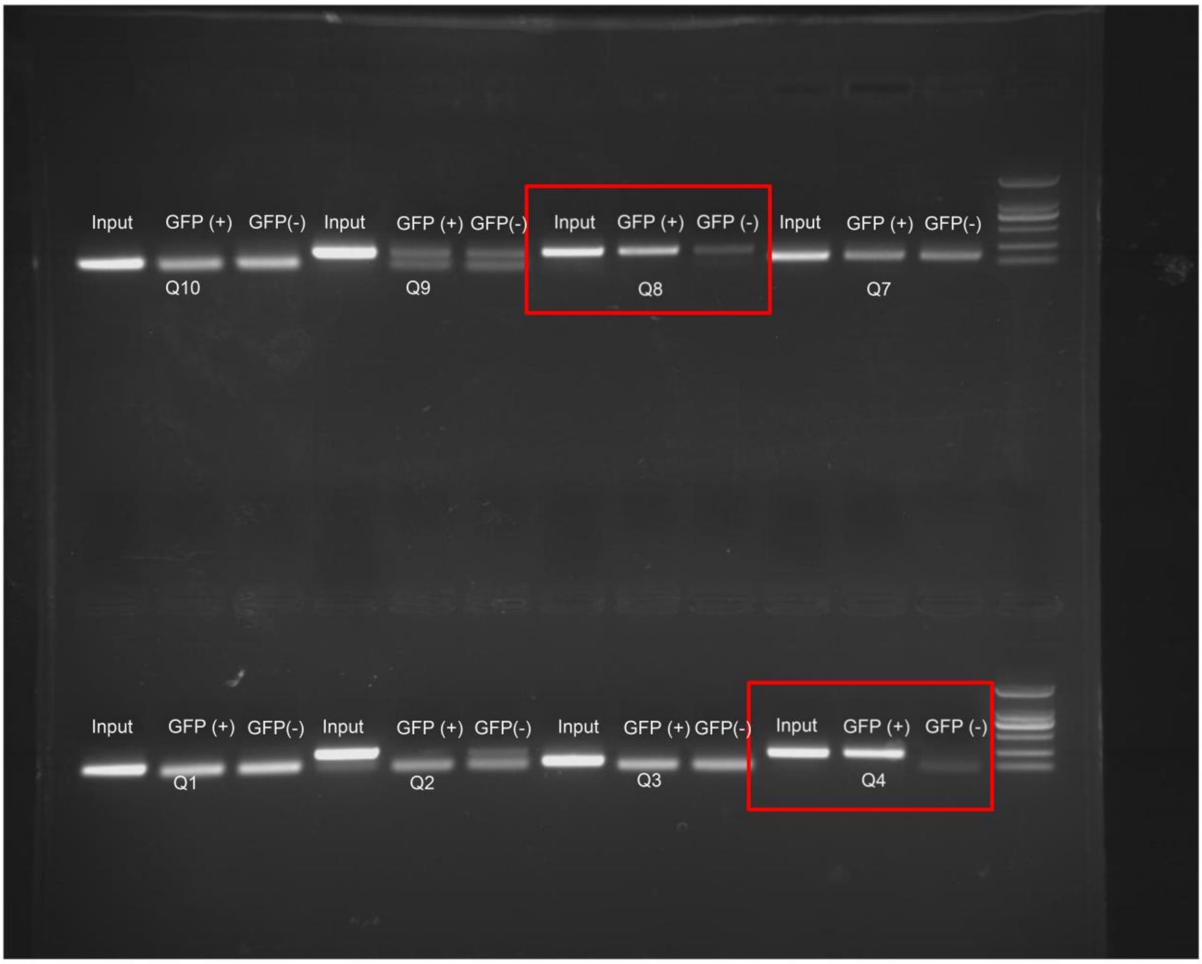

Figure S5. NLP7 binds to the promoter of *NRT1.1* in *vivo*. ChIP assays were performed with *nlp7-1* seedlings expressing pNLP7:NLP7-GFP fusion protein grown on 10mM ammonium nitrate medium for 7d. A sample without antibody was used as the negative control for the ChIP-PCR. Q4 and Q8 in the red boxes are the regions showing the binding activity of NLP7 to *NRT1.1* promoter shown in Fig.5.

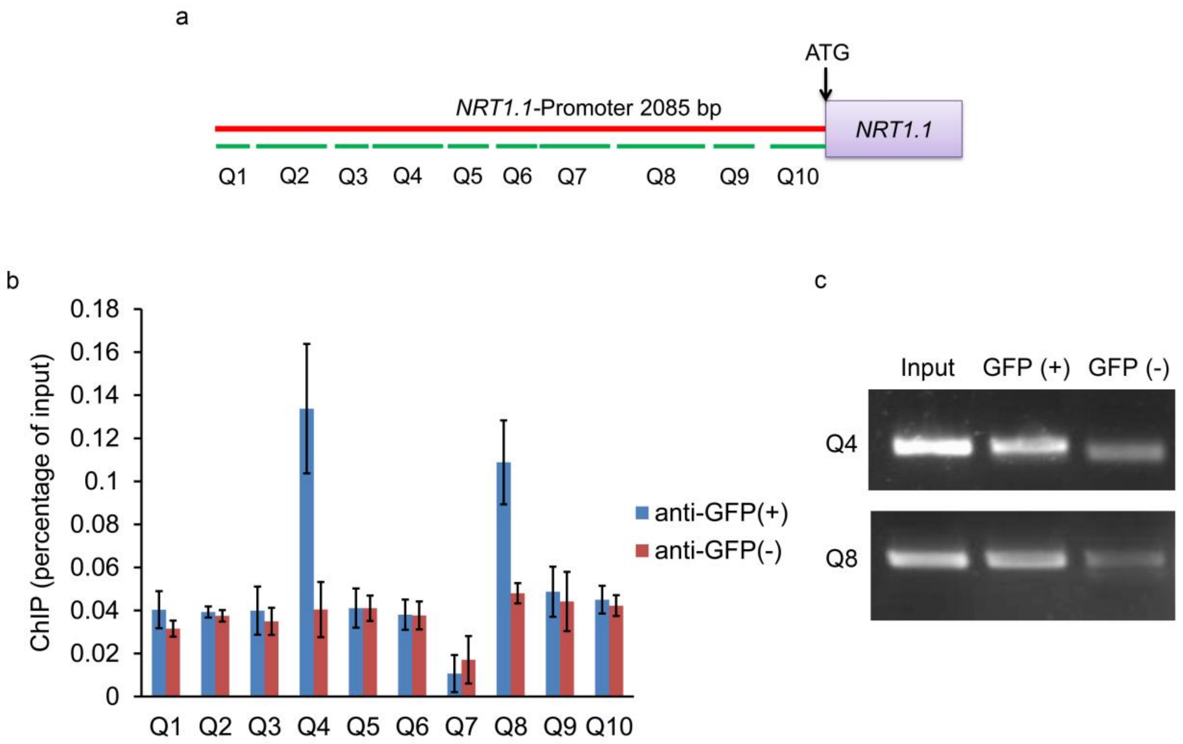

Figure S6. NLP7 binds to the promoter of *NRT1.1* in *vivo*. (a) Segmentation schematic diagram of the promoter of *NRT1.1*. Q1-Q10 represent different fragments of *NRT1.1* promoter. (b) ChIP-qPCR assays show that NLP7 binds to *NRT1.1* promoter in *vivo*. ChIP assays were performed with *nlp7-1* seedlings expressing p35S::NLP7-GFP fusion protein grown on 10mM ammonium nitrate medium for 7d. A sample without antibody was used as the negative control for the ChIP-qPCR (n=3). (c) ChIP-PCR assays prove that NLP7 binds to *NRT1.1* promoter in *vivo*. ChIP assays were performed with the same seedlings as in (b). Input was used as positive control and a sample without antibody was used as the negative control.

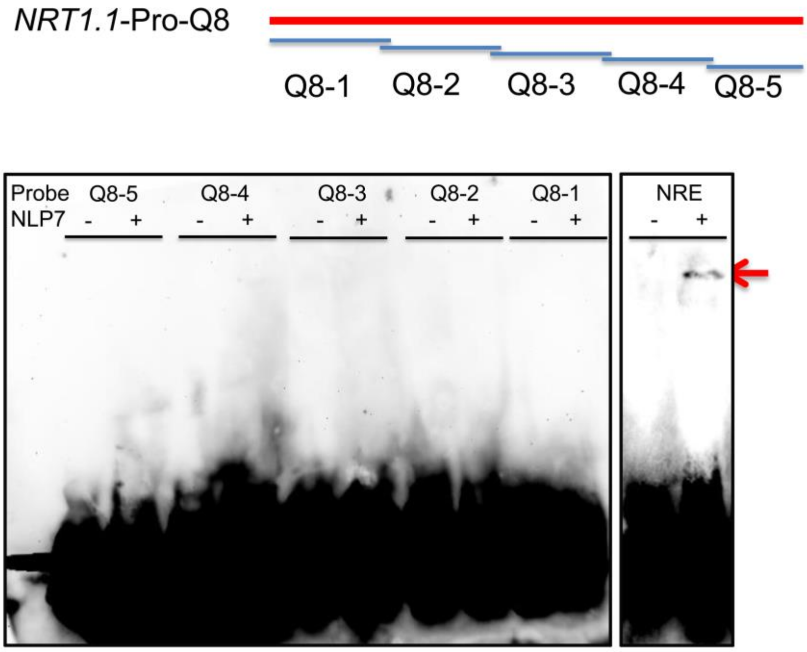

Figure S7. NLP7 cannot bind to the Q8 region of *NRT1.1* promoter in *vivo*. EMSA assay was performed to investigate the binding of NLP7 to Q8 region of *NRT1.1* promoter. Red arrow indicated the positions of protein-DNA complexes caused by binding of NLP7 to DNA probe. The NRE probe was used as a positive control. The DNA probes were listed in Supplementary Dataset 6.

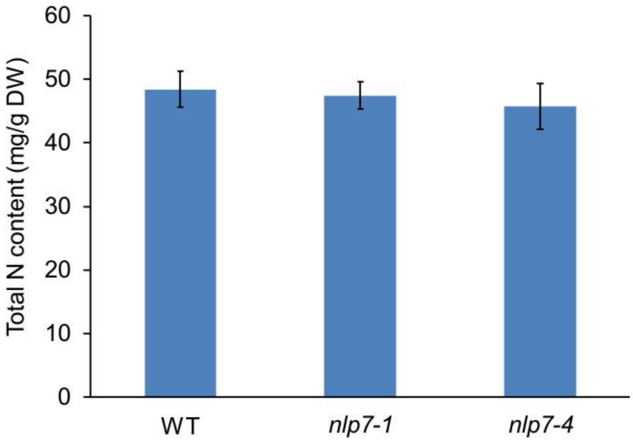

Figure S8. The total nitrogen content in *nlp7* mutants was similar to wild type. Seedlings grown on 10mM ammonium nitrate medium for 7d were collected for total nitrogen content test. Error bars mean ± SD of four biological replicates.
